# Estimating the yield stability of heat-tolerant rice genotypes under various heat conditions across reproductive stages: a 5-year case study

**DOI:** 10.1038/s41598-021-93079-x

**Published:** 2021-06-30

**Authors:** Chao Wu, Kehui Cui, Qian Li, Liuyong Li, Wencheng Wang, Qiuqian Hu, Yanfeng Ding, Ganghua Li, Shah Fahad, Jianliang Huang, Lixiao Nie, Shaobing Peng

**Affiliations:** 1 Guangxi Key Laboratory of Functional Phytochemicals Research and Utilization, Guangxi Institute of Botany, Guangxi Zhuang Autonomous Region and Chinese Academy of Sciences, Guilin, 541006 China; 2 National Key Laboratory of Crop Genetic Improvement, MOA Key Laboratory of Crop Ecophysiology and Farming System in the Middle Reaches of the Yangtze River, Huazhong Agricultural University, Wuhan, 430070 Hubei China; 3 College of Agronomy, Nanjing Agricultural University, Key Laboratory of Crop Physiology, Ecology, and Production Management, Ministry of Agriculture, Jiangsu Collaborative Innovation Center for Modern Crop Production, Nanjing, China; 4 Hainan Key Laboratory for Sustainable Utilization of Tropical Bioresource, College of Tropical Crops, Hainan University, Haikou, 570228 China; 5 Department of Agronomy, The University of Haripur, Haripur, 22620 Pakistan

**Keywords:** Abiotic, Heat

## Abstract

Heat events during the reproductive stages of rice plants induce great yield losses. Cultivating heat-tolerant varieties is a promising strategy for guaranteeing grain security under global warming scenarios. Most heat-tolerant rice genotypes were identified under heat during the flowering stage, but it is unclear whether these currently screened heat-tolerant rice genotypes maintain stable high grain yields when heat stress occurs during the other reproductive stages. In the present study, two notable heat-tolerant rice cultivars, Nagina22 and Shanyou63, and one typical heat-sensitive cultivar, Liangyoupeijiu, were evaluated for their yield response and yield stability under heat treatments during the panicle initiation, flowering, and grain filling stages during 2010–2014. Our results revealed that rice cultivars respond differently to heat stress during different reproductive stages. Nagina22 was the most tolerant to heat stress during the flowering and grain filling stages but was susceptible during panicle initiation; Shanyou63 was the most tolerant to heat stress during panicle initiation and grain filling and was moderately tolerant to heat stress during the flowering stages. Genotype and genotype-by-environment interaction biplot yield analysis revealed that Shanyou63 exhibited the highest stability in high grain yield, followed by Nagina22, and Liangyoupeijiu exhibited stable low grain yield when experiencing heat stress across the three reproductive stages. Our results indicate that the heat tolerance of different rice cultivars depends on the reproductive stage during which heat stress occurs, and the effects manifest as reductions in grain yields and seed setting rates. Future efforts to develop heat-tolerant varieties should strive to breed varieties that are comprehensively tolerant to heat stress during any reproductive stage to cope with the unpredictable occurrence of future heat events.

## Introduction

Climate change, triggered by human activity and characterized by global warming^1^, is increasingly threatening grain production and food security. Rice is a staple food for approximately half of the world’s population, but frequent extreme heat events have taken a toll on rice production^2^. An analysis of historical data concluded that rice grain yields decrease by 14% for every 1 °C increase in average daily temperature^3^ and 10% for every 1 °C increase in average nighttime temperature^4^. Additionally, high nighttime temperature was reported to have more serious impact on rice grain yield than high daytime temperature treatment under certain heat-stressed conditions^4^.

Rice plants are highly susceptible to heat stress during their reproductive stages^5,6^, i.e., from panicle initiation to complete panicle maturity^7^. Heat events may occur at any stage during the reproductive phase of rice under global warming conditions. Rice grain yield responds differently to heat stress with different underlying mechanisms during different reproductive stages^5^. Heat stress during panicle initiation induces morphological abnormalities in the reproductive organs, which results in reductions in the number of spikelets per panicle, grain weight and seed setting rate^5^; heat stress during flowering induces physiological abnormalities in the male organs^8^, thus reducing the seed setting rate^9^; and heat stress during grain filling disturbs carbohydrate metabolism, which reduces grain weight^10^. To cope with the unpredictable occurrence of heat events during the reproductive stages, rice plants should develop corresponding unique mechanisms for heat mitigation and adaptation.

The development of heat-tolerant rice varieties is a critical strategy for coping with global warming. Heat stress during flowering represents a greater threat to rice grain yields than heat stress during other growth stages^11^. Thus, most of the heat-tolerant rice genotypes were screened under heat during the flowering stage^12^. The rice cultivar Nagina22, which is reported to have high spikelet fertility (80%) even when experiencing heat stress as high as 38 °C during flowering^13^, is well known as a heat tolerant cultivar and widely used in breeding programmes for heat tolerance^14,15^. The rice variety Shanyou63, a rice hybrid famous for its high yield and broad adaptability in China^16^, has also been reported to achieve high spikelet fertility under heat stress in controlled and field conditions during the flowering stage^17,18^.

In recent years, heat events have occurred in the middle and lower reaches of the Yangtze River, which constitutes one of the main paddy rice production areas in China, as early as mid-July, during which panicle development of midseason rice occurs. However, the heat tolerance of typical heat-tolerant rice cultivars during nonflowering reproductive stages has rarely been studied^6^. Future heat event occurrence is becoming unpredictable and serious, and it is uncertain whether the existing heat-tolerant rice genotypes identified during certain reproductive stages could resist heat stress during other critical reproductive stages. Evaluations of rice grain yields and their determinants under heat stress during different stages of reproductive growth enable a comprehensive understanding of the overall heat tolerance of different rice cultivars.

The objective of this study was to (i) evaluate the variation in grain yield and yield components in response to heat stress in two typical heat-tolerant cultivars, Nagina22 and Shanyou63, during panicle initiation, flowering, and grain filling and (ii) assess their overall heat tolerance by evaluating the yield stability of the two typical heat-tolerant cultivars based on the average yield response to heat stress across different reproductive stages.

## Results

### High temperature treatments

In temperature-controlled facilities during 2010, the recorded mean daytime/nighttime temperatures were 33.5 °C/31.1 °C under the high nighttime temperature (HNT) treatment and 36.6 °C/27.9 °C under high daytime temperature (HDT) treatment imposed during the panicle initiation stage, which were approximately 0.0 °C/3.2 °C and 3.1 °C/0.0 °C higher than those of the control (CK) treatment (daytime/nighttime: 33.5 °C/27.9 °C), respectively; during the flowering stage, the mean daytime/nighttime temperatures were 32.7 °C/28.4 °C and 34.3 °C/26.6 °C under the HNT and HDT temperature treatments, which were approximately 0.0 °C/1.8 °C and 1.6 °C/0 °C higher than those of the CK treatment, respectively; during the grain filling stage, the mean daytime/nighttime temperatures were 29.2 °C/26.2 °C and 30.7 °C/23.7 °C under the HNT and HDT temperature treatments, which were approximately 0.0 °C/2.5 °C and 1.5 °C/0.0 °C higher than those of the CK treatment, respectively (Table 1).

**Table 1:** Average temperatures (daytime/nighttime, °C) and average relative humidity (%) in the greenhouse from 2010–2014.

Year	Treatment	Reproductive stages		
		Panicle initiation	Flowering	Grain filling
2010	CK	33.5/27.9	32.7/26.6	29.2/23.7
	HNT	33.5/31.1	32.7/28.4	29.2/26.2
	HDT	36.6/27.9	34.3/26.6	30.7/23.7
	HDNT	–	–	–
2011	CK	31.5/26.5^A^ (93.4)^B^	31.0/26.0 (85.5)	30.6/25.6 (83.1)
	HNT	31.6/29.4 (82.4)	31.7/28.7 (83.6)	30.5/28.8 (78.4)
	HDT	34.9/26.4 (81.9)	33.5/26.3 (77.4)	33.3/25.7 (79.8)
	HDNT	35.3/29.8 (80.5)	33.3/28.4 (80.4)	33.3/29.7 (80.5)
2012	CK	–	30.5/26.4 (80.8)	30.0/25.9 (81.6)
	HNT	–	31.8/32.1 (75.7)	31.6/32.3 (72.5)
	HDT	–	33.6/28.7 (78.6)	34.2/27.5 (75.9)
	HDNT	–	34.6/30.9 (78.0)	35.4/31.1 (74.6)
2013	CK	31.9/27.2 (80.2)	–	–
	HNT	33.5/31.9 (74.0)	–	–
	HDT	36.1/26.7 (81.5)	–	–
	HDNT	38.3/31.5 (75.2)	–	–
2014	CK	28.9/26.4 (85.8)	28.1/26.7 (86.2)	26.1/25.9 (80.8)
	HNT	–	–	–
	HDT	34.3/26.7 (80.7)	34.5/26.6 (83.1)	34.5/26.3 (75.0)
	HDNT	–	–	–

HNT, high nighttime temperature treatment; HDT, high daytime temperature treatment; HDNT, high daytime and nighttime temperature treatment; CK, control. A, average temperature records of daytime and nighttime during the five experimental years; B, values in the brackets were average relative humidity during the whole day.

In 2011 during the panicle initiation stage, the recorded mean daytime/nighttime temperatures were 31.6 °C/29.4 °C, 34.9 °C/26.4 °C and 35.3 °C/29.8 °C under the HNT, HDT and high daytime and nighttime temperature (HDNT) treatments, which were approximately 0.1 °C/2.9 °C, 3.4 °C/0.0 °C and 3.8 °C/3.3 °C higher than those of the CK treatment (daytime/nighttime: 31.5 °C/26.5 °C), respectively; during the flowering stage, the mean daytime/nighttime temperatures under the heat treatments were 31.7 °C/28.7 °C, 33.5 °C/26.3 °C and 33.3 °C/28.4 °C under the HNT, HDT and HDNT temperature treatments, which were approximately 0.7 °C/2.7 °C, 2.5 °C/0.3 °C and 2.3 °C/2.4 °C higher than those of the CK treatment, respectively; during the grain filling stage, the mean daytime/nighttime temperatures were 30.5 °C/28.8 °C, 33.3 °C/25.7 °C and 33.3 °C/29.7 °C under the HNT, HDT and HDNT temperature treatments, which were approximately 0.0 °C/3.2 °C, 2.7 °C/0.1 °C and 2.7 °C/4.1 °C higher than those of the CK treatment, respectively (Table 1).

In 2012, during the flowering stage, the recorded mean daytime/nighttime temperatures were 31.8 °C/32.1 °C, 33.6 °C/28.7 °C and 34.6 °C/30.9 °C under the HNT, HDT and HDNT temperature treatments, which were approximately 1.3 °C/5.7 °C, 3.1 °C/2.3 °C and 4.1 °C/4.5 °C higher than those of the CK treatment (daytime/nighttime: 30.5 °C/26.4 °C), respectively; during the grain filling stage, the mean daytime/nighttime temperatures were 31.6 °C/32.3 °C, 34.2 °C/27.5 °C and 35.4 °C/31.1 °C under the HNT, HDT and HDNT temperature treatments, which were approximately 1.6 °C/6.4 °C, 4.2 °C/1.6 °C and 5 °C higher than those of the CK, respectively (Table 1).

In 2013, during the panicle initiation stage, the recorded mean daytime/nighttime temperatures were 33.5 °C/31.9 °C, 36.1 °C/26.7 °C and 38.3 °C/31.5 °C under the HNT, HDT and HDNT temperature treatments, which were approximately 1.6 °C/4.7 °C, 4.2 °C/0.0 °C and 6.4 °C/4.3 °C higher than those of the CK treatment (daytime/nighttime: 31.9 °C/27.2 °C), respectively (Table 1).

In 2014, during the panicle initiation stage, the recorded mean daytime/nighttime temperatures were 34.3 °C/26.7 °C under the HDT temperature treatments, which were approximately 5.4 °C/0.3 °C higher than those of the CK treatment (daytime/nighttime: 28.9 °C/26.4 °C), respectively; during the flowering stage, the mean daytime/nighttime temperatures were 34.5 °C/26.6 °C under the HDT temperature treatments, which were approximately 6.4 °C/0.0 °C higher than those of the CK treatment, respectively; during the grain filling stage, the mean daytime/nighttime temperatures were 34.5 °C/26.3 °C under the HDT temperature treatments, which were approximately 8.4 °C/0.4 °C higher than those of the CK treatment, respectively (Table 1).

### Responses of rice grain yield and yield components to heat treatments

Heat treatments during the reproductive stages reduced rice grain yield and yield components. Significant differences existed among the three reproductive stages in grain yield (*P* < 0.05), seed setting rate (*P* < 0.05), spikelet number (*P* < 0.01), and grain weight (*P* < 0.01), among the three cultivars in grain yield (*P* < 0.05) and spikelet number (*P* < 0.01), and among the three heat treatments in grain weight (*P* < 0.01). No significant interactions were found among the reproductive stages, cultivars and heat treatments except for grain weight, which was significantly affected by (*P* < 0.01) the interaction between reproductive stage and cultivar/heat treatment (Table 2). On average, heat treatments during the panicle initiation, flowering and grain filling stages reduced the grain yield by 49.2%, 25.0% and 16.4% in Nagina22, by 19.5%, 34.1% and 16.5% in Shanyou63, and by 40.0%, 55.9% and 28.7% in Liangyoupeijiu, respectively, across the HNT, HDT, and HDNT treatments.

**Table 2:** Relative yield and yield components (%) under heat treatments.

Cultivar	Growth stage	Temperature treatment	Grain yield	Seed setting rate	Spikelet number	Panicle number	Grain weight
Nagina22	Panicle initiation	HNT	64.7	80.3	93.2	91.7	96.0
		HDT	48.9	66.9	80.1	107.0	89.6
		HDNT	38.7	60.1	83.7	90.5	84.2
		Mean	50.8cd	69.1abc	85.7bc	96.4ab	89.9f
	Flowering	HNT	84.7	94.4	95.8	89.7	101.1
		HDT	71.3	83.7	98.5	88.8	97.8
		HDNT	69.1	88.4	89.2	88.1	100.0
		Mean	75.0ab	88.8ab	94.5ab	88.8b	99.6a
	Grain filling	HNT	80.2	86.7	97.8	93.2	95.9
		HDT	86.7	91.9	99.5	98.5	96.1
		HDNT	83.9	92.0	97.1	98.2	93.2
		Mean	83.6a	90.2a	98.1a	96.6ab	95.1cd
Shanyou63	Panicle initiation	HNT	78.1	79.5	97.0	98.6	97.8
		HDT	72.2	87.4	94.7	96.1	93.8
		HDNT	91.2	97.8	98.3	103.4	92.4
		Mean	80.5ab	88.3ab	96.7a	99.4a	94.6de
	Flowering	HNT	78.7	77.6	105.0	99.2	98.2
		HDT	54.6	55.8	97.5	98.6	98.6
		HDNT	64.4	62.6	101.8	99.2	98.7
		Mean	65.9abc	65.3bc	101.4a	99.0a	98.5ab
	Grain filling	HNT	91.9	93.0	103.4	94.5	99.0
		HDT	76.9	76.3	98.5	101.8	94.5
		HDNT	81.9	79.1	100.6	102.7	95.6
		Mean	83.5a	82.8ab	100.8a	99.7a	96.4bcd
Liangyoupeijiu	Panicle initiation	HNT	62.6	78.1	84.2	97.5	94.1
		HDT	68.3	97.4	72.5	99.7	93.8
		HDNT	49.1	68.9	73.5	97.6	87.4
		Mean	60.0bcd	81.4ab	76.7c	98.2a	91.8ef
	Flowering	HNT	53.3	58.3	90.8	96.6	97.0
		HDT	39.7	41.7	98.6	101.3	97.2
		HDNT	39.2	43.2	95.2	101.2	95.9
		Mean	44.1d	47.7c	94.9ab	99.7a	96.7bcd
	Grain filling	HNT	79.4	82.7	95.3	101.6	99.2
		HDT	68.8	71.0	106.5	97.7	97.3
		HDNT	65.8	69.0	101.2	102.4	96.0
		Mean	71.3abc	74.2ab	100.9a	100.6a	97.5abc
Stage (S)			4.70*	2.73*	12.98**	0.82	27.36**
Cultivar (C)			4.32*	2.44	5.87**	2.97	2.05
Heat treatment (T)			1.56	0.69	0.38	0.83	9.48**
S × C			2.17	2.61*	2.35	0.67	3.39*
S × T			0.21	0.45	0.98	0.28	2.92*
C × T			0.45	0.24	0.56	0.61	0.86
S × T × C			0.31	0.38	0.59	0.72	0.79

HNT, high nighttime temperature treatment; HDT, high daytime temperature treatment; HDNT, high daytime and nighttime temperature treatment. *, significance at 5%; **, significance at 1%

The seed setting rate decreased by an average of 30.9%, 11.2% and 9.8% in Nagina22, 11.7%, 34.7% and 17.2% in Shanyou63, and 18.6%, 52.3% and 25.8% in Liangyoupeijiu under heat stress during the panicle initiation, flowering and grain filling stages, respectively, across the three heat treatments (Table 2). The spikelet number decreased by an average of 14.3%, 5.5% and 1.9% in Nagina22, by 3.3%, − 1.4% and − 0.8% in Shanyou63, and by 23%, 5.1% and − 0.9% in Liangyoupeijiu when heat stress was imposed during the panicle initiation, flowering and grain filling stages, respectively, across the three heat treatments. Grain weight decreased by an average of 10.1%, 0.4% and 4.9% in Nagina22, by 5.4%, 1.5% and 3.6% in Shanyou63, and by 8.2%, 0.8% and 2.5% in Liangyoupeijiu under heat stress during the panicle initiation, flowering and grain filling stages, respectively, across the three heat treatments. Heat stresses had negligible effects on panicle number in the three rice cultivars (Nagina22, Shanyou63 and Liangyoupeijiu) during the three reproductive stages (Table 2).

### Average yield response and yield stability of different rice cultivars under heat stress

**Figure 1:**
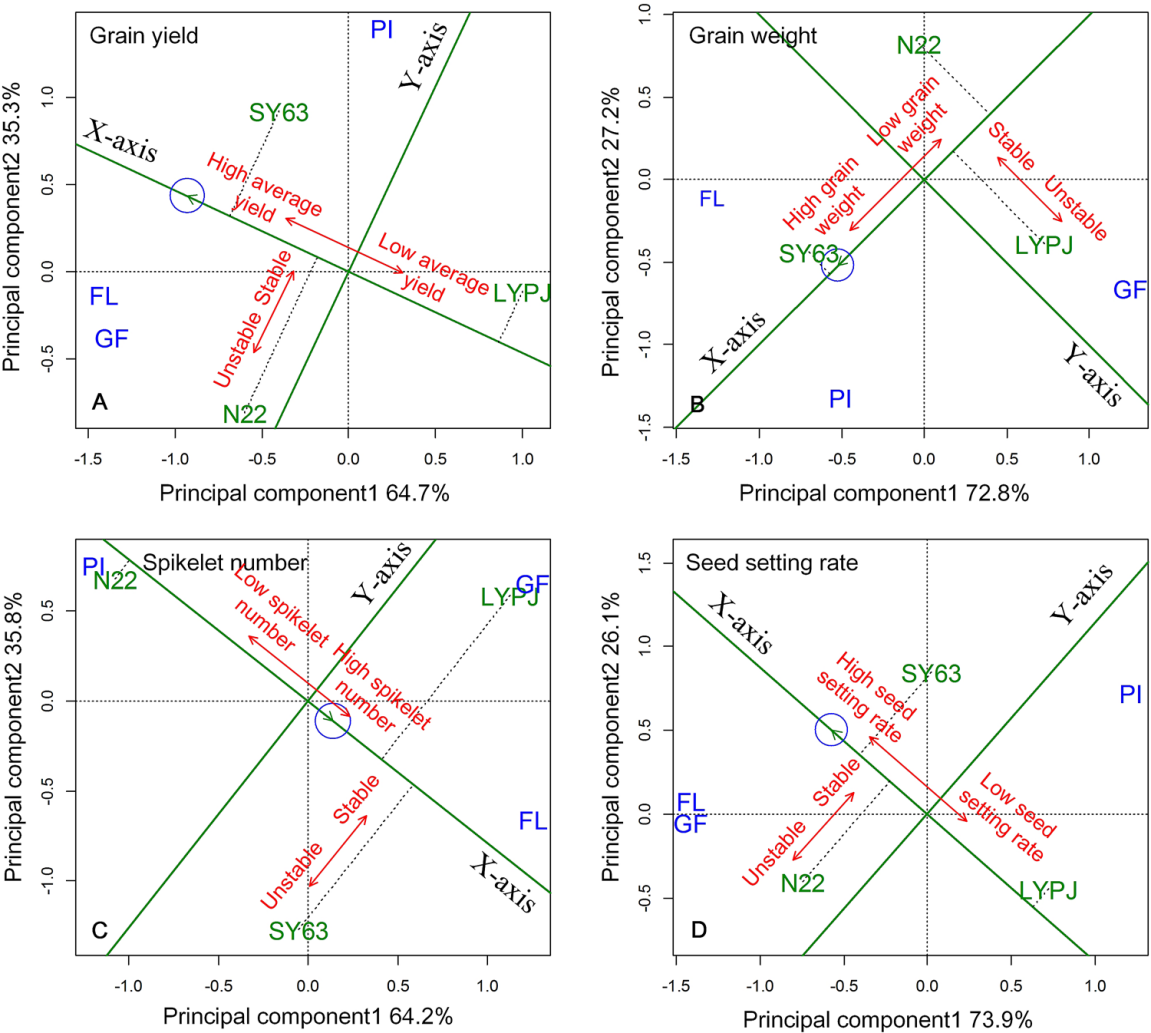
GGE biplot view of mean relative grain yields and yield components vs. stability of these yield characteristics for the three rice cultivars tested over 5 years. PI, panicle initiation stage; FL, flowering stage; GF, grain filling stage; SY63, Shanyou63; N22, Nagina22; LYPJ, Liangyoupeijiu.

Figure 1 shows the results view of mean relative grain yields and yield components vs. stability of these yield characteristics by genotype and genotype by environment interaction biplot analysis. The averages of relative grain yields and relative yield components across heat treatments and experimental years for the three rice cultivars were approximated by the projections (indicated by dotted line) of their markers onto the average environmental coordinate (AEC). The double arrow that runs parallel to the *x-axis* indicates the direction of yield performance of the different cultivars. Shanyou63 maintained the highest average grain yield under heat stress during the three reproductive stages, followed by Nagina22 and Liangyoupeijiu (Fig. 1A). Each cultivar’s resilience to heat stress (stability) was estimated by its projection (dotted line) on the AEC *y-axis.* The double arrow that parallels the *y-axis* shows the ranks of the different cultivars with respect to the stability of their yield or yield components under heat stress. The greater the absolute length of the projection for grain yield or yield components of a given cultivar, the less stable the grain yield or yield components. Based on this analysis, Shanyou63 and Liangyoupeijiu were more stable in terms of average grain yield than Nagina22 under heat stress during all three reproductive stages (Fig. 1A). Shanyou63 experienced the highest average grain weight, spikelet number, and seed setting rate under heat stress during the three reproductive stages (Fig. 1). Shanyou63 also exhibited the highest stability in grain weight, followed by Nagina22 (Fig. 1B). Nagina22 was the most stable in terms of spikelet number, followed by Shanyou63 (Fig. 1C). Liangyoupeijiu had the most stable seed setting rate (Fig. 1D).

## Discussion

Rice plants are vulnerable to heat stress during the reproductive stages. The average daytime temperatures of the heat treatments (HDT, HDNT) were approximately 3.1–5.4 °C, 1.6–6.4 °C and 1.5–8.4 °C higher than those of the control during the panicle initiation, flowering, and grain filling stages, respectively, from 2010  to 2014. The average nighttime temperatures under the heat treatments (HNT, HDNT) were approximately 3.2–4.7 °C, 1.8–5.7 °C and 2.5–6.4 °C higher than those under the control during panicle initiation, flowering, and grain filling, respectively, across the 5 experimental years (Table 1). The critical temperatures during the reproductive stages of panicle initiation, flowering, and grain filling for rice development are 33.1 °C, 35.0 °C and 31.3 °C, respectively^5,6,19^. In the present study, the recoded temperatures (Table 1) in the temperature-controlled facilities approached the critical temperature thresholds of rice plants during the three reproductive stages during 2010–2014 and thus were considered heat stress.

### Responses of grain yields and yield components to heat stress during different reproductive stages

The current study investigated the responses of yield and yield components of three rice cultivars to heat stress imposed during three stages of reproductive growth (panicle initiation, flowering, and grain filling). Reductions in grain yields induced by heat treatments during panicle initiation were due to synchronous reductions in seed setting rate, spikelet number, and grain weight; however, yield reductions were primarily due to reduced seed setting rates that resulted from heat treatments during the flowering and grain filling stages (Table 2). These findings are supported by previous research showing that heat stress significantly reduced seed setting, spikelets per panicle, and grain weight during panicle initiation^20^ but only reduced seed setting significantly during flowering and grain filling^21^. Shi et al.^22^ also reported that heat stress at different reproductive stages (gametogenesis, anthesis) impacted grain yields and yield components to different degrees. Together, these data indicate that grain yield and yield components respond to heat stress differently depending on the reproductive stage during which the stress occurs.

The seed setting rate was reduced by heat stress during all three reproductive stages in all three cultivars, with the most serious reduction occurring when heat stress was applied during flowering (32.7% on average), followed by panicle initiation (20.4% on average) and grain filling (17.6% on average) (Table 2). Similarly, a previous study reported that rice plants were most sensitive to heat stress during flowering^11^. Decreased seed setting rates induced by heat stress during flowering have been attributed to physiological abnormalities in the reproductive organs, e.g., inhibition of anther dehiscence caused by a disturbance in water metabolism, reduction in pollen shedding due to disruption of anther dehydration, impaired stigma receptivity attributed to reduced stigma peroxidase and stigma-surface esterase activity, and reduced pollen germination caused by ion imbalance, disturbance in carbohydrate metabolism and changes in the levels of regulators such as phytohormones under heat stress^5^; however, decreased seed setting under heat stress during panicle initiation might be due to morphological abnormalities of the reproductive organs, including panicle enclosure, structural abnormalities in the anthers, disruptions in the function of the septum and tapetum, inhibition of microsporogenesis, morphological abnormalities of the stigma and pollen, etc.^5,15,23^.

During the grain filling stage that follows flowering stage, heat treatments impairs spikelet fertility (such as insufficient pollen grain on stigma^18^ and exserted stigmas^15^; In the viewpoint of assimilate supply, heat treatment also inhibits the assimilate production via lowering photosynthetic rate^24^ and promoting senescence of functional leaves^25^, and further reduces the assimilate distribution to grains; heat exposure may also impair early embryo^26^ and seed development^27^; additionally, heat stress often show adverse effects on activities of enzymes for starch synthesis^28^. These adverse influences of heat stress may partly explain the reduced seed setting rate during the grain filling stage.

Grain weight was reduced by heat treatment during panicle initiation and grain filling. Notably, the negative impact of heat stress on grain weight during the panicle initiation stage (average 7.9% reduction) seemed to be more severe than that during the grain filling stage (average 3.7% reduction) in the present study (Table 2). In the 2014 season experiment, the average temperatures during panicle initiation (daytime/nighttime: 34.3 °C/26.7 °C) were nearly the same as those during grain filling (daytime/nighttime: 34.5 °C/26.3 °C) (Table 1), and the average relative grain weight (90.2 ± 3.2%, avg. ± SD) under heat stress during panicle initiation was lower than the average relative grain weight (96.3 ± 2.0%) during the grain filling stage in the three studied rice cultivars (Supplemental Table S1). Similarly, larger reductions in grain weight were observed during panicle initiation than during the grain filling stage under the same high soil temperature regime^29^.

Rice grain weight is determined by multiplying grain size by grain plumpness. Previous studies have reported reductions in grain weight induced by heat stress during grain filling^21^. The lighter grain weight of heat-stressed plants has been primarily attributed to impaired grain plumpness, which is associated with a shorter duration of grain filling and an altered grain filling rate^26^. While the grain filling rate is increased by moderately high temperatures^21^, it is also decreased by extremely high temperatures^25^; however, moderate and extremely high temperatures also shorten the duration of grain filling and thus reduce the final grain weight. In contrast, the decreased grain weight caused by heat stress during panicle initiation has primarily been attributed to decreased grain size, which is associated with reduced nonstructural carbohydrates^26^, undeveloped vascular bundles^30^, and reduced grain size (length and width), which are involved in disrupted formation of meristems^31^. Therefore, our results and previous investigations together suggest that heat stress during the reproductive phase impairs grain weight; heat stress during panicle initiation detrimentally affects the development of spikelets and filling of grains. The underlying mechanisms of the severe effects of heat injury on grain weight during the panicle initiation stage should be further investigated.

The early reproductive phase is crucial for panicle development because it is strongly associated with spikelet number per panicle. The spikelet number was reduced by heat treatments only during panicle initiation in this study (Table 2). Previous studies also reported reduced panicle size in heat-stressed rice plants^20,32^ and that heat stress attenuated the differentiation of secondary branches and attached florets while promoting degradation of branches and attached florets^31^. The fewer spikelets per panicle, branches and attached florets in heat-stressed rice plants were associated with inhibited transportation and enhanced degradation of cytokinins induced by heat treatment during the panicle initiation stage^5,33^. It is noteworthy that the heat treatments during flowering and grain filling had no substantial effects on spikelet number per panicle in this study (Table 2). Taken together, grain yield and yield components (seed setting rate, spikelet number, and grain weight) responded differently to heat stress with different underlying mechanisms during different reproductive stages in rice cultivars.

### Cultivar differences in response to heat stress during different reproductive stages

In the current study, we found that Nagina22 generally retained the highest relative grain yields (75.0–83.6%) among the three cultivars under heat treatments during flowering and grain filling (Table 2); however, this cultivar produced the lowest relative grain yields (50.8%) among the three cultivars under heat treatments during panicle initiation (Table 2). In previous investigations, heat treatments during panicle initiation^31^ or booting^34^ induced serious spikelet sterility in Nagina22. Additionally, our results indicate that Nagina22 experienced lower seed setting (69.1%) and grain yields (50.8%) under heat treatment during panicle initiation than during flowering (88.8% for seed setting rate and 75.0% for grain yield) or during grain filling (90.2% and 83.6%), respectively (Table 2). These data suggest that Nagina22 is highly tolerant to heat stress during flowering and grain filling but is susceptible to heat stress during panicle initiation. Our results reveal that different rice cultivars might have different heat resistance depending on the reproductive stage during which heat stress occurs and that vulnerability to heat stress manifests as changes in grain yields and the seed setting rate.

In the current study, the rice cultivar Shanyou63 exhibited high relative grain yields under heat stress during panicle initiation (80.5%) and grain filling (83.5%) and moderate relative grain yields during flowering (65.9%) (Table 2). However, Liangyoupeijiu consistently produced low relative grain yields (60% for panicle initiation, 44.1% for flowering, and 71.3% for grain filling) under heat stress during all reproductive stages (Table 2). Previous studies reported similar findings for both Shanyou63 and Liangyoupeijiu^17,31^. The mean vs. stability view of the genotype and genotype by environment interaction biplot indicates that Shanyou63 had the highest average yield, followed by Nagina22 and Liangyoupeijiu; Shanyou63 also demonstrated higher stability than Nagina22 in terms of grain yield (Fig. 1A), grain weight (Fig. 1B), and seed setting rate (Fig. 1D) under heat stress. Li et al.^35^ reported that Shanyou63 exhibited higher relative grain yields than Nagina22 under in-season heat stress. Thus, these data demonstrate that Shanyou63 might consistently achieve relatively high grain yields under heat stress during the three reproductive phases. Moreover, Shanyou63 is considered a milestone in the development and production of hybrid rice varieties in China due to its high yield and wide adaptability to various environments; it is therefore commonly used in both basic research and agronomic studies related to biotic/abiotic stress tolerance^16^. In conclusion, Shanyou63 exhibited higher comprehensive tolerance to heat stress than Nagina22 throughout the reproductive period (Table 3), supported by its high and stable relative grain yields under heat stress during different reproductive stages.

**Table 3:** Ideal rice genotypes with comprehensive tolerance to heat stress.

	Panicle initiation	Flowering	Grain filling	Entire reproductive phase
Liangyoupeijiu	−	−	−	−
Nagina22	−	++	++	+
Shanyou63	+ +	+	++	++
Ideal heat tolerant genotype	** ++ **	** ++ **	** ++ **	** ++ **

−, heat sensitive; +, moderately heat tolerant; ++, heat tolerant. + in bold font indicates the more tolerant to heat stress than the rice cultivar Nagina22/Shanyou63.

**Figure 2:**
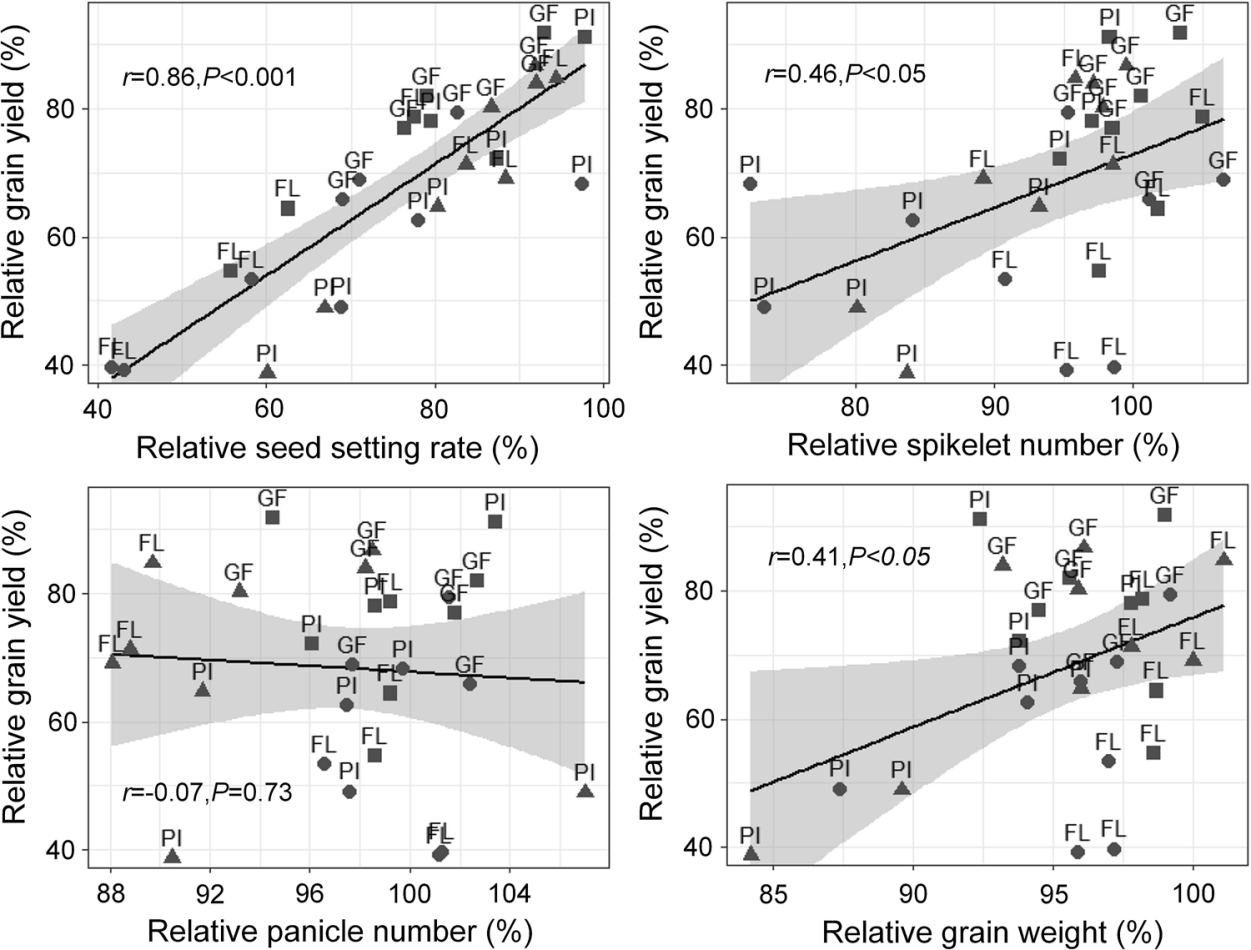
Relationship between relative grain yield and relative yield components. PI, panicle initiation stage; FL, flowering stage; GF, grain filling stage; filled circle, Liangyoupeijiu; filled triangle, Nagina22; filled square, Shanyou63.*, significant correlation at the *p* < 0.05 level; ns, no significant correlation.

Yield variation in rice genotypes was due mainly to the differences in seed setting rate under heat as supported by the facts that (i) seed setting rate suffered from the most serious impairment under heat treatments during the reproductive stages (Table 2), and (ii) the relative seed setting rate correlated significantly with relative grain yield (Fig. 2). During the panicle initiation stage, the heat-induced reduction in seed setting rate in Nagina22 and Liangyoupeijiu was largely due to incomplete panicle enclosure, inhibited anther dehiscence and pollen shedding, reduced pollen vigor, which were ascribed to the reduced levels of indole-acetic acid and gibberellin acid under heat treatments; heat-tolerant Shanyou63 maintained stable levels of indole-acetic acid and gibberellin acid, and thus generated complete panicle exsertion and small reductions in pollen vigor, anther dehiscence, and pollen shedding^31^. During the flowering stage, the reduction in seed setting rate in heat-sensitive Liangyoupeijiu was largely due to the reduced anther dehiscence, pollen viability and pollen germination under heat treatments; the high seed setting rate in Nagina22 and Shanyou63 was attributed to the good performance in anther dehiscence, pollen shedding, and pollen germination under heat treatments^36,37^. During the grain filling stage, heat treatment reduced grain plumpness especially in inferior grains in heat-sensitive Liangyoupeijiu, thus decreased seed setting rate and grain weight^38,39^; however, heat treatments had small effects on grain plumpness in Nagina22 and Shanyou63^16,40,41^.

### Perspectives on heat tolerant cultivars under unpredictable heat events

Heat injury varies not only with the intensity, duration and fluctuation of temperatures above optimal limits but also with the stage of plant development^5^. The timing and duration of heat events, which threaten crop production, are expected to become more problematic in large parts of the world, making it necessary to explore all aspects of plant growth and development to breed, modify and select crops adapted to such conditions. We proposed that identification and screening for ideal heat-tolerant genotypes that exhibited comprehensive heat tolerance to heat events during any growth stage is an effective strategy for coping with the increasing unpredictability of future heat stress events. The rice cultivar Shanyou63 is currently a near model cultivar of this objective (Table 3).

Most studies have assessed the heat tolerance of rice cultivars only during a specific period, usually during flowering^6,12^. The rice cultivar Nagina22, which has drawn special attention for its extreme tolerance to heat stress during the flowering stage, has been widely used in studies of the physiological and molecular mechanisms of heat tolerance^13^. The quantitative trait locus (QTL) *qHTSF4.1* on chromosome 4 in Nagina22 was found to be responsible for high seed setting rates under heat stress during the flowering stage^42^. It was proposed that the transfer of heat tolerance QTLs from Nagina22 to varieties could maintain stable grain yields at high temperatures during flowering by molecular breeding approaches^43^.

However, *qHTSF4.1,* which controls spikelet fertility under heat stress, was identified in Nagina22 during the flowering stage. We speculated that *qHTSF4.1* could arouse certain mechanisms to overcome the physiological abnormalities in anthers that induced spikelet sterility under heat stress during flowering, but it is not clear if the improved genotypes carrying *qHTSF4.1* could cope with heat stress during other reproductive stages, such as panicle initiation, during which heat induces morphological abnormalities in the reproductive organs. In the present study, the widely recognized typical rice cultivar Nagina22 was proven to be susceptible to heat treatment during the panicle initiation stage (Table 3). Thus, the QTL *qHTSF4.1,* which is linked to heat tolerance during flowering, and other tolerance donors that could overcome heat stress during the other critical reproductive stages should be integrated to produce ideal heat-tolerant genotypes (Table 3) that are comprehensively tolerant to heat across the reproductive phase.

## Conclusions

Our results indicate that grain yield losses during heat stress are primarily due to decreased seed setting rates, and also spikelet numbers and grain weights, which vary depending on the rice cultivar and the reproductive stage during which heat stress occurs. Nagina22, a heat-tolerant rice cultivar, was most tolerant during the mid-late reproductive phase (flowering and grain filling) but was highly susceptible during the early reproductive phase (panicle initiation). The rice cultivar Shanyou63, which is comprehensively tolerant to heat stress during panicle initiation, flowering, and grain filling, could currently represent an ideal cultivar with comprehensive tolerance to heat stress. Future efforts to breed heat-tolerant varieties should focus on increasing comprehensive tolerance to heat stress throughout the entire reproductive phase to cope with the unpredictability of future heat stress events.

## Materials and methods

Two typical heat-tolerant rice cultivars (Nagina22 from the Genetics Resource Center of the International Rice Research Institute, Shanyou63, an elite rice hybrid produced by the Sanming Institute of Agricultural Sciences, Sanming City, China) and one heat-sensitive rice cultivar (Liangyoupeijiu, a high-yielding variety produced by the Jiangsu Academy of Agricultural Sciences, Nanjing City, China) were used during 2010–2014^35,44,45^. To have all of the materials arrive at the same developmental phase of panicle initiation and flowering, the sowing dates of the three rice cultivars were adjusted according to records of their growth phases in our previous studies. Seeds were sown in plastic seeding trays with loamy soil after dormancy was broken at 50 °C for 5 days. At the three-leaf stage, four seedlings of each cultivar were transplanted into a 14.0 L plastic pot (height, 28.5 cm, top diameter, 30.0 cm; base diameter, 25.0 cm) containing a mixture of 17.0 kg soil (loam: sand, 2:1) and 12.5 g compound fertilizer (N:P_2_O_5_:K_2_O, 16%:16%:16%). The potted rice plants were randomly arranged with four replications under natural ambient conditions in each experimental year. Seedlings were thinned to three plants per pot (each plant had three tillers) 8 days after transplanting, and the main stems were tagged. A total of 1.0 g of urea was top dressed per pot 10 days after transplanting. The plants were irrigated with a water table depth of approximately 2 cm from transplanting to maturity. Each pot was manually rotated clockwise by 90° every 7 days to avoid positional effects. Pests, diseases, birds, and weeds were intensively controlled.

We assessed the effects of heat stress on rice grain yield and other yield components, including seed setting, spikelet number, grain weight, and panicle number, using 5-year experiments at the same site. The experiments were conducted in plastic-covered sheds in 2010^45^ and in greenhouses from 2011–2014^35,44^ at Huazhong Agricultural University, Wuhan, China (30°29ʹN, 114°22ʹE). In each greenhouse, a dehumidifier, an air conditioner, two ventilators and two sensors for monitoring the air temperature and relative humidity were running automatically to maintain the temperatures in the greenhouse at the targeted setting.

Global climate warming exhibits strong diurnal variations, characterized as diurnal asymmetric warming^46^. The effects of HDT and HNT and HDNT treatments on rice cultivars were thus examined in the present study. The experimental design included four temperature treatments: HNT treatment (high nighttime temperature from 19.00–07.00 h), HDT treatment (high daytime temperature from 07.00–19.00 h), HDNT treatment (high daytime and nighttime temperature for 24 h), and a CK treatment, in which rice plants were grown under favorable temperatures during the entire day (the same temperature regimes as the HNT treatment) and night (the same temperature regimes as the HDT treatment).

For the CK treatment, the temperatures were set at 24 °C from 05:00 to 06:00; 31 °C from 10:00 to 11:00; 32 °C from 12:00 to 13:00; and 27 °C from 20:00 to 21:00 when the temperature treatment was imposed during the panicle initiation stage and were set at 24 °C from 05:00 to 06:00; 30 °C from 10:00 to 11:00; 31 °C from 12:00 to 13:00; and 27 °C from 20:00 to 21:00 when the temperature treatment was imposed during the flowering/grain filling stage.

For HNT, the temperatures were set at 31 °C from 05:00 to 06:00; at 31 °C from 10:00 to 11:00; at 32 °C from 12:00 to 13:00; and 32 °C from 20:00 to 21:00 when the temperature treatment was imposed during the panicle initiation stage and were set at 28 °C from 05:00 to 06:00; 30 °C from 10:00 to 11:00; 31 °C from 12:00 to 13:00; and 31 °C from 20:00 to 21:00 when the temperature treatment was imposed during the flowering/grain filling stage.

For HDT treatment, the temperatures were set at 24 °C from 05:00 to 06:00; at 38 °C from 10:00 to 11:00; at 39 °C from 12:00 to 13:00; and 27 °C from 20:00 to 21:00 when the temperature treatment was imposed during the panicle initiation stage and were set at 24 °C from 05:00 to 06:00; 34 °C from 10:00 to 11:00; 35 °C from 12:00 to 13:00; and 27 °C from 20:00 to 21:00 when the temperature treatment was imposed during the flowering/grain filling stage.

For HDNT treatment, the temperatures were set at 31 °C from 05:00 to 06:00; at 38 °C from 10:00 to 11:00; at 39 °C from 12:00 to 13:00; and 32 °C from 20:00 to 21:00 when the temperature treatment was imposed during the panicle initiation stage and were set at 28 °C from 05:00 to 06:00; 34 °C from 10:00 to 11:00; 35 °C from 12:00 to 13:00; and 31 °C from 20:00 to 21:00 for the temperature treatment imposed during the flowering/grain filling stage.

In the controlled greenhouses, the relative humidity (%) was set to 80%, and the air temperature and relative humidity were controlled with a central auto controller (Auto Greenhouse Monitoring and Data Management System, Version 3.00, Auto, Beijing, China), with the temperatures inside the greenhouses being gradually changed to approach the next set of temperature values. The air temperature and relative humidity were recorded 5 cm above the rice canopy using a stand-alone sensor (HOBO, H08-003-02, Onset Computer Corporation, Bourne, MA, USA).

The average temperatures and average relative humidity recorded in the temperature-controlled facilities during the experiments are shown in Table 1, part of which were reported in previous studies^35,44,45^. With few exceptions, all temperature treatments were implemented during panicle initiation in 2010, 2011, 2013, and 2014 and during flowering and grain filling in 2010, 2011, 2012, and 2014. The HNT treatment was not implemented in 2014, and the HDNT treatment was not included in 2010 and 2014. Heat treatments were not implemented during the panicle initiation stage in 2012 and were not implemented during flowering and grain filling in 2013 (Table 1). The recordings of relative humidity during 2011–2014 were presented in Table 1; however, the relative humidity was not available during 2010.

All plants were carefully cultivated under natural ambient conditions, after which they were moved to temperature-controlled facilities at the start of panicle initiation (when the first row of floral primordia was visible on the shoot apex, the stage can be referred to R0 stage according to Counce et al.^7^), flowering (when an average of 5% spikelets of the main tillers had exserted their anthers, the stage can be referred to R3 stage according to Counce et al.^7^), and grain filling (when an average of 95% spikelets of the main tillers had exserted their anthers, the stage can be referred to R5 stage according to Counce et al.^7^) to undergo temperature treatments for 15, 7, and 30 days, respectively, during each rice-growing season. To determine the grain yield and yield components, the whole plants for each biological replicate in each experimental year were harvested at maturity except for 2013, in which three panicles of the main stems were harvested, and then threshed manually to determine the grain yield and yield components.

Panicles per plant was counted before harvest. After harvest, salt water (1.12 g cm^−3^ density) was used to separate filled grains from empty and unfilled grains. The grains were dried, and partially filled grains were picked out carefully by manually pressing the grains between the forefinger and thumb. Filled grains were oven dried at 80 °C to a constant weight to determine grain yield and grain weight (mg grain^−1^). Spikelet number was defined as all spikelets per panicle, the seed setting rate was calculated as 100 × the ratio of the number of fully filled spikelets to the total number of spikelets per panicle in the five-year experiments, and grain yield was defined as yield per plant in the five years except for 2013, during which grain yield was defined as yield per main stem, as reported in our previous studies^35,44,45^.

Relative values of grain yield and yield components (%) for different rice cultivars were used to evaluate cultivar-specific responses to heat treatments in this study. Relative values were defined as the ratio of a value under a given heat treatment to the value under the control treatment for grain yield or yield components in the same rice cultivar. Relative values of grain yield and yield components (Supplemental Table S1) were calculated according to the absolute values of rice grain yield and yield components (Supplemental Table S2) obtained from 2010 to 2014 by master/doctorate research performed by our group^35,44,45^.

In the present study, the five experimental years were considered replications. The average values of relative yield and yield components (%) are presented in Table 2 under specific temperature treatments across the experimental years. The mean relative values of yield and yield components under the different temperature treatments for all experimental years were used for analysis of variance, regression analysis, and for genotype and genotype-by-environment interaction biplot analysis, which was used to visually assess any genotype × high temperature interactive effects on a given relative yield trait across experimental years and heat treatments during panicle initiation, flowering, and grain filling and to rank genotypes according to stability of their yield and yield components under the heat treatments. The GGEBiplotGUI package in R statistical software (ver. 3.4.1 Analytical Software, Tallahassee, FL, USA) was used according to Bernal and Villardon^47^, and the R code for the analysis of multivariate stability (genotype and genotype-by-environment interaction biplots) was used according to Frutos et al.^48^.

## Supplementary Information

Supplementary Information.
